# Climate change impacts on crop breeding: Targeting interacting biotic and abiotic stresses for wheat improvement

**DOI:** 10.1002/tpg2.20365

**Published:** 2023-07-06

**Authors:** Carlos A. Robles‐Zazueta, Leonardo A. Crespo‐Herrera, Francisco J. Piñera‐Chavez, Carolina Rivera‐Amado, Gudbjorg I. Aradottir

**Affiliations:** ^1^ Global Wheat Program, International Maize and Wheat Improvement Center (CIMMYT) Texcoco México; ^2^ Mamoré Research and Innovation Limited Harpenden UK

## Abstract

Wheat (*Triticum aestivum* L.) as a staple crop is closely interwoven into the development of modern society. Its influence on culture and economic development is global. Recent instability in wheat markets has demonstrated its importance in guaranteeing food security across national borders. Climate change threatens food security as it interacts with a multitude of factors impacting wheat production. The challenge needs to be addressed with a multidisciplinary perspective delivered across research, private, and government sectors. Many experimental studies have identified the major biotic and abiotic stresses impacting wheat production, but fewer have addressed the combinations of stresses that occur simultaneously or sequentially during the wheat growth cycle. Here, we argue that biotic and abiotic stress interactions, and the genetics and genomics underlying them, have been insufficiently addressed by the crop science community. We propose this as a reason for the limited transfer of practical and feasible climate adaptation knowledge from research projects into routine farming practice. To address this gap, we propose that novel methodology integration can align large volumes of data available from crop breeding programs with increasingly cheaper omics tools to predict wheat performance under different climate change scenarios. Underlying this is our proposal that breeders design and deliver future wheat ideotypes based on new or enhanced understanding of the genetic and physiological processes that are triggered when wheat is subjected to combinations of stresses. By defining this to a trait and/or genetic level, new insights can be made for yield improvement under future climate conditions.

## Core Ideas

- To improve wheat yield, it is critical to understand the responses to co‐occurring biotic and abiotic stresses.
- The use of omics and data‐driven approaches will facilitate the integration of complex traits in breeding programs.
- Developing wheat ideotypes will require multidisciplinary work across stakeholders from researchers to end users.

**Abbreviations**
AgMIP: Agricultural Model Intercomparison and Improvement Project
eCO_2_
: elevated CO_2_ atmospheric concentration
G × E: genotype by environment interaction
G × E × M: genotype by environment by management interaction
HTP: high‐throughput phenotyping
RUE: radiation use efficiency

## 1 PLANTS EXPERIENCE MULTIPLE SIMULTANEOUS ABIOTIC AND BIOTIC STRESSES

Wheat (*Triticum aestivum* L.) is an important commodity, with ∼22% of wheat produced traded internationally (Shewry & Hey, 2015). Shifts in climatic conditions over time are likely to cause further spatiotemporal variations in abiotic and biotic stresses for wheat producers that will likely lead to significant spatial changes in yield and grain quality (Fradgley et al., 2022). These challenges will need to be addressed from multiple perspectives, including research‐based solutions. Too often, current crop research efforts operate in silos of distinct disciplines that do not reflect the plant‐ or crop‐level experience of stress: encountering multiple abiotic and biotic stressors simultaneously and sequentially during field growth seasons (Suzuki et al., 2014). We propose that this is an important research gap in the development of climate change adaptation strategies for crops, limiting opportunities to breed future crops that integrate resilience and potential for higher production.

Conservative predictions for a 2°C increase in global mean temperature (Deutsch et al., 2018) are predicted to change the timing and severity of both biotic and abiotic stresses as well as changing or expanding their geographic distribution (IPCC Secretariat, 2021). This includes expected changes in pest survival rates during heat waves or frosts, increased latency of spores for fungi, shifts in population growth dynamics and geographical distribution for insects, and reduction of crop cycle length (Atkinson & Urwin, 2012; Eigenbrode & Macfadyen, 2017). To avoid further damage to the environment, wheat productivity must increase without expanding agricultural land area and limiting the use of water and fertilizers. The reduction of inputs being enforced through legislative changes in several countries in the world, stricter regulations on permissible pesticides and changes in usage of growth promoters is expected to have a confounding effect on the likelihood of significant crop damage and resultant food insecurity (O'Hanlon, 2019).

To increase crop resilience to changing climates, it is proposed that selecting and crossing parents with improved resource utilization and allocation within the canopy will be key for increasing yields (Burgess et al., 2022; Kromdijk & Long, 2016). This will have little impact on crop productivity if adaptation to harsher heat and drought conditions (Langridge & Reynolds, 2021), increases in atmospheric CO_2_ concentration (Ainsworth & Long, 2021), and co‐occurring biotic and abiotic stressors (Juroszek & von Tiedemann, 2013) are not considered.

Wheat varieties mostly represent germplasm adapted to biotic and abiotic stressors selected and assessed at a point in time with specific climate conditions. Wheat ideotypes can be selected for their responses to certain environmental conditions (i.e., genotype × environment, G × E) or alternatively with superior performance across targeted environments (i.e., stable genotypes with low G × E). This represents a major gap in current research, and further understanding of the real‐time and dynamic genetic and physiological mechanisms involved in perceiving and responding to different and co‐occurring or sequential stresses is urgently required.

Here, we discuss common biotic and abiotic stresses in wheat, the effect of their interactions on productivity and highlight the necessary transfer of knowledge from the laboratory to the field. We also describe existing tools to help breeders deliver future wheat ideotypes and propose new approaches to address interacting biotic and abiotic stresses in crop research.

**Core Ideas**

- To improve wheat yield, it is critical to understand the responses to co‐occurring biotic and abiotic stresses.
- The use of omics and data‐driven approaches will facilitate the integration of complex traits in breeding programs.
- Developing wheat ideotypes will require multidisciplinary work across stakeholders from researchers to end users.

## 2 THE MULTIPLYING EFFECT OF INTERACTING BIOTIC AND ABIOTIC STRESSES

Global wheat production faces a range of interacting biotic and abiotic stresses. Water stress is one of the most important factors affecting wheat productivity and is usually more severe when high temperatures or prolonged drought occur. Water and heat stress, both individually and in combination, are known to cause a reduction in stomatal conductance (*gs*), spike fertility, lower photosynthetic capacity and efficiency, and affect the source‐sink balance (Posch et al., 2022) ultimately reducing yield.

Heat stress is known to alter wheat plant architecture, and if aggravated by drought, it causes impaired osmotic adjustment, damage by reactive chemicals (e.g., reactive oxygen species) and reduces photosynthetic pigment content (Barnabás et al., 2008; Tricker et al., 2018). Drought and heat stress are often intertwined and can take place during the same crop cycle. For example, drought might be the result of a heat wave at a local level or due to chronic increases in regional temperatures, which can cause severe or lethal effects at the organ or plant level, and by increasing evaporation rates and depleting water stored in the soil (Breshears et al., 2021). Crops have developed adaptations to drought such as the reduction of amino acid content (Michaletti et al., 2018), or the extra accumulation of sugars to be remobilized to the grain when photosynthesis cannot support grain filling (Guo et al., 2018; Marček et al., 2019; Nemati et al., 2018).

Low nitrogen (N) availability can also co‐exist with drought and heat stress disturbing phloem transport, changing sap composition (Sevanto, 2014) and depleting reserves of water‐soluble carbohydrates in plant leaves and stems. A recent study found that drought stress reduces protein content needed for photosynthetic activity especially Rubisco and light‐harvesting complexes such as cyclophilin 38, HCF136, and adenosine triphosphate synthase. Additionally, Rubisco activase is down‐modulated inhibiting photosynthesis in drought‐stressed leaves (Michaletti et al., 2018). When heat stress is applied to wheat, metabolite content gets altered in the different plant organs. In the spikes, glycine, methionine, maltose, and raffinose content increases while aspartate decreases; in the stems, glutamine and β‐alanine content decreases (Impa et al., 2019). Negative effects of N deficiency in the soil are exacerbated by drought‐driven decline in soil microbiota that makes inorganic N forms unavailable for the plant, causing growth impairment and floret sterility, altering the source‐sink balance and leading to a rapid decline in the amino‐acid content of the phloem (Caputo & Barneix, 1997; Curci et al., 2017; Jeuffroy & Bouchard, 1999). The latter examples may trigger plant defense mechanisms or alter resource allocation in different ways to cope with interaction of different stresses; hence, it is important to understand wheat physiological and genetic responses to develop integrative understanding of these interactions.

Atmospheric elevated CO_2_ concentration (eCO_2_) studies in wheat are scarce compared to other crops (e.g., rice, soybean, maize, and tobacco), but scarcer are field experiments where more than one abiotic stress interacts with eCO_2_. Understanding of these interactions is limited since the majority of eCO_2_ studies have been conducted in controlled conditions which differ fundamentally from how light and N are distributed within the canopy in the field, as well as ignoring soil physiochemical conditions and farmer management practices (Cormier et al., 2016; Poorter et al., 2016).

Some findings indicate that when air temperature increases, eCO_2_ can stimulate greater wheat biomass accumulation and plant height with up to ∼53% yield increment compared to regular ambient CO_2_ (Fitzgerald et al., 2016). However, grain quality decreases when the C:N ratio increases (Robinson et al., 2012) and when there are reductions in grain protein content due to eCO_2_ (Taub et al., 2008). Other reported yield reductions when eCO_2_ combines with heat and/or drought stress conditions include inhibition of nitrate assimilation (Bloom et al., 2010), higher photosynthetic rates (Bloom et al., 2010; Chavan et al., 2019), and photo assimilate depletion (Chavan et al., 2019). Additionally, eCO_2_ can co–occur with pest outbreaks (e.g., *Zymoseptoria tritici* Desm. and *Fusarium graminearum* Petch.) reducing yield even further (Váry et al., 2015). Hence, we will need to design future studies more objectively by systematically targeting and generating data on the magnitude of these interactions (e.g., drought × eCO_2_, increased temperature × eCO_2_, pathogen × drought × eCO_2_, pathogen × drought × increased temperature × eCO_2_) in field conditions.

Life cycles of wheat pathogens and pests are influenced by their environment, and many are known to thrive at elevated temperatures. Wheat pathogen response to changes in environmental conditions include changes in geographical distribution, seasonal phenology, and population dynamics. Studies of wheat leaf rust (*Puccinia triticina* Eriks.) in Europe suggest warmer field conditions increase inoculum accumulation during winter and spring and extend the duration of latency period of spores (Miedaner & Juroszek, 2021). Further, modeling scenarios for Ethiopia have shown that stripe rust (*Puccinia striiformis* f. sp. *tritici*) resistant wheat varieties can have higher yield than susceptible ones under drought conditions (Abro et al., 2017). It has also been shown that there are genotypic differences in the response of heat stress and susceptibility to yellow rust in bread wheat in Egypt suggesting that heat stress tolerance could be bred alongside rust resistance and not penalize yield (Megahed et al., 2022).

For cereal aphids, a temperature rise of 2°C would increase the number of generations reproduced per year in regions where temperatures are currently near optimal for aphid development (Aljaryian & Kumar, 2016; Deutsch et al., 2018; Hullé et al., 2010). With rising temperatures, it is expected that pests and pathogens will disperse beyond their original habitats; for example, the English grain aphid (*Sitobion avenae* Fabricius) has already extended its habitat to cover almost the entirety of the North Hemisphere (Bebber et al., 2013). However, for an insect pest to become invasive in new geographical areas, external factors such as the presence and quality of the host, food trade, and agricultural practices play an important role (Bebber et al., 2014; Skendžić et al., 2021). Nonetheless, climate sets the dispersal range of pests to the point at which a pest can become invasive, increase earlier infestations of native pests, and/or increase the presence or fecundity of multiple pests in wheat growing regions.

Interesting effects on co‐occurring biotic and abiotic stresses, such as the relation of climate‐driven changes in phloem chemistry and aphid feeding patterns, highlight the need for an integrated approach to study interacting stresses. For example, in cereals, the greenbug (*Schizaphis graminum* Rondani) has better reproductive rates when its plant host does not suffer from drought stress (Cabrera et al., 1995; Pendleton & Veerabomma, 2008; Sumner et al., 1986) because the nutritional value of the phloem sap is correlated to the level of aphid infestation (Douglas & van Emden, 2007). When *S. graminum* is exposed to drought stress, its feeding rate increases due to low quality phloem sap, and this combination of biotic and abiotic stresses reduces photosynthetic capacity (Dorschner et al., 1986; Ryan et al., 1987). Additionally, studies have shown that the concentration of phenylalanine, proline, glutamine, and tryptophan increases in the plant when fed upon by *S. graminum* (Sandström et al., 2000; Zhang et al., 2019). The concentration of these amino acids also increases in drought‐susceptible wheat genotypes (Michaletti et al., 2018), which highlights the importance of incorporating aphid resistance in drought‐tolerant wheat varieties. These effects must also be taken into consideration when making plant selection decisions: drought stressed plants may have reduced insect damage, but this may be an artifact of the abiotic stress interaction causing low sap nutritional value in drought‐stressed plants.

Irrigated and high rainfall wheat growing regions are commonly affected by collapsed crop fields (lodging) that result in low yield and quality. Future shifts in weather conditions will cause further spatiotemporal changes that will strongly affect crop lodging incidence and expansion in its geographical distribution. Greater susceptibility to pests and diseases is common in crops subjected to lodging events (Berry et al., 2004; Joseph et al., 2020), but lodging can also result from foot‐rot or root‐rot diseases and insect‐attacked stems (Pinthus, 1974). Fusarium foot‐rot (*Fusarium* spp.), eyespot or strawbreaker (*Pseudocercosporella herpotrichoides* (Fron) Deighton), Hessian fly (*Mayetida destructor* Say), and sawflies (*Cephus cinctus* Norton and *Cephus pygmaeus* L.) are the most common diseases and pests that can cause lodging (Berry et al., 2004; Bockus et al., 2010; Pinthus, 1974). It is evident that interactions, and the changing magnitude of lodging, disease, and pest damage should be more proactively addressed by research and breeding programs.

In the case of stem sawfly, plant damage and lodging are caused by larval tunneling down to the base of the stems (Oiestad et al., 2017) destroying parenchyma tissue and vascular bundles. This induces low kernel number, inefficient harvest (due to the collapsed crop), and loss of grain quality (Beres et al., 2007). Breeding for solid stems has been targeted over the past 60–70 years to reduce sawfly‐related losses (Varella et al., 2016). Interestingly, solid stems can also increase mechanical strength (Bisht et al., 2022), which is crucial to improve lodging resistance in wheat and could be an important source of carbohydrates reserve during grain filling. This showcases the opportunity to solve two problems with a single strategy for example, through the development and application of reliable markers for solid stems (Oiestad et al., 2017; Piñera‐Chavez et al., 2021).

The examples discussed in this section demonstrate the necessity to not only consider individual biotic and abiotic stresses but to understand and integrate their current as well as future interactions. Developing methods to assess the genetic and physiological mechanisms underlying these interactions will help to accelerate deployment of knowledge and ensure the successful adaptation of wheat to climate change. For this to be achieved, it is necessary to develop novel controlled environment and field‐based studies that consolidate this new knowledge with current and future breeding efforts.

## 3 UNDERSTANDING INTERACTING STRESSES: FROM LAB TO FIELD

Innovation has been a key driver in increasing food availability and affordability, especially in the developed world (Campos, 2021). Primary research will be a key basis for some of the major innovations required to adapt wheat to the multiple challenges of a changing climate if they can be translated into knowledge applied at scale in farmers’ fields.

Large environmental datasets are becoming available, including daily solar radiation, air temperature, and relative humidity from multiple field locations. These datasets can be used to characterize target environments, used as covariates for statistical analyses and applied to derive crop parameters with modeling (referred to as enviromics) (Costa‐Neto et al., 2021). Remote sensing data collected at different scales can give us information on the physiological status of the crop (phenomics), and increasingly cheaper DNA‐based technologies can help us unravel the basis of traits with a large amount of genetic information (genomics). New data types that profile plant chemical response processes can also be harnessed (metabolomics) as can real‐time expression of genes and/or functional proteins (transcriptomics and proteomics). These datasets can be integrated through the utilization of newly developed statistical machine learning models to predict the performance of unobserved plant genotypes.

Controlled experiments often do not provide an accurate picture of the biotic and abiotic stresses that field‐grown plants experience (Poorter et al., 2016). This means that results may not be directly applicable in a current or future breeding context. To address this, we need to make more efficient use of field and laboratory high‐throughput phenotyping (HTP) capabilities and combine conventional and HTP to develop statistical models that could be used to introduce complex trait enhancements in photosynthesis, *gs*, biomass accumulation, and radiation use efficiency (RUE) into breeding pipelines. It has been shown that *gs* can be predicted with 97% accuracy using machine learning models (Gibbs et al., 2021), and RUE and photosynthesis can be predicted with 69% (Robles‐Zazueta et al., 2021) and 48% accuracy (Robles‐Zazueta et al., 2022), respectively. These models need to be fine‐tuned to improve accuracy, especially for gas exchange by increasing the amount of ground truth data available to build the models. Nonetheless, the time spent collecting data in the field and processing samples in the lab can be reduced approximately by 27 times for agronomic traits and 40 times for photosynthetic traits (Robles‐Zazueta et al., 2021, 2022). HTP protocols applied in combination with interdisciplinary efforts such as the Agricultural Model Intercomparison and Improvement Project (AgMIP) (Rosenzweig et al., 2013) can aid in the development of low‐cost platforms to phenotype multiple traits at the same time in the field, and coupled with field informed controlled environment experiments, combined stresses can be studied at a mechanistic and genetic level.

There are several parametric, semi‐parametric, and non‐parametric statistical models based on machine learning available for genomic prediction (Crossa et al., 2021). This includes Bayesian methods to estimate the genomic best linear unbiased predictors incorporating relationship genomic matrices with environmental covariables to assess G × E, predicting unobserved genotypes in different years or site‐years (Jarquín et al., 2014). Kernel functions capture nonlinear patterns from original input data using machine learning algorithms in the transformed data for genomic prediction values of complex breeding traits and their interactions (i.e., G × E, G × G, G × G × E, G × E × M). Furthermore, partial least squares regression modeling has been used to predict single‐ and multi‐trait performance dividing the site years datasets for training and validation of the models (Montesinos‐López et al., 2022a,b). Finally, deep learning neural networks have been used to capture small cryptic associations between markers that reflect genes and genetic epistasis (Montesinos‐López et al., 2019, 2021). We propose that all these integrative methods offer new opportunities to combine data on multiple co‐occurring or sequential stresses and predict crop performance more dynamically and accurately than using conventional methods and individual protocols or analysis timepoints.

Beyond genetic effects, metabolomics can be a powerful technology to accelerate the understanding of metabolic pathways related to abiotic and biotic stress tolerances and interactions (Razzaq et al., 2021). Together with metabolomics, proteins play a key role in the plant responses to pathogens, and proteomics has enabled the identification of germplasm and genetic response to stem sawfly and *Fusarium*‐related diseases (Biyiklioglu et al., 2018; Feussner & Polle, 2015; Gunnaiah et al., 2012; Kage et al., 2017). Metabolomics and proteomics can offer a deeper understanding of stress interactions by providing information on additional levels of regulation and defining the role of small molecules while increasing the spatiotemporal resolution of the interaction analysis (Feussner & Polle, 2015). Likewise, understanding gene expression over time can augment the resolution of traditional genetic and genomic data.

These available tools can help elucidate the complex processes that affect wheat growth and underpin current and future productivity. A concerted effort to develop a platform to integrate these and develop an intuitive user interface to enable wide uptake would be a key step change in mainstreaming this integrative approach. Attention should be given to developing this as an open access solution considering low‐resource programs without access to state‐of‐the‐art omics technologies or computational resources, or the economic ability to sow thousands of lines in the field to explore the physical interaction of the biotic and abiotic stresses mentioned above.

## 4 FUTURE PERSPECTIVES

The global wheat system from single plant to global commodity is fascinating yet complex. Wheat scientists are continually challenged to develop new ideotypes based on changing target environments with overlapping and interacting stress factors. We argue that delivery of resilient wheat production now and, in the future, requires a holistic approach integrating not just yield and quality components, but an integrated view of plant architecture, physiology, biotic and abiotic stresses affecting the crop system. New open access experimental and data platforms are needed to support cutting‐edge science efficiently translated into the field to understand the interacting stresses. This will ensure that information flows from research to farmers faster. The use of omics, new integrative statistical and computational methods, and crop modeling will allow researchers and breeders to simulate experimental and breeding systems for hundreds or thousands of genotypes, multiple environments and years to predict new ideotypes and understand the underlying genetic and physiological processes triggered when wheat is subjected to multiple stresses.

We conclude that none of these efforts will have true meaning unless their main goal is to benefit farmers, improve the sustainability of crop production, and enhance food security. Ultimately, any research project should include tangible impacts that shifts from the current siloed approach toward a more holistic package of wheat resilience (and for any crop) to climate change. This will create a fertile ground for a second Green Revolution with a smooth transition from large‐scale industrial agricultural production to a more resilient and sustainable production which includes traits recommended from national programs for a specific region creating lasting positive impacts for society and the environment.

## AUTHOR CONTRIBUTIONS

**Carlos A. Robles‐Zazueta**: Conceptualization; writing—original draft; writing—review and editing. **Leonardo A. Crespo‐Herrera**: Conceptualization; writing—review and editing. **Francisco J. Piñera‐Chavez**: Conceptualization; writing—review and editing. **Carolina Rivera‐Amado**: Conceptualization; writing—review and editing. **Gudbjorg I. Aradottir**: Conceptualization; writing—original draft; writing—review and editing.

## CONFLICT OF INTEREST STATEMENT

The authors declare no conflict of interest.
